# Antimicrobial use on *Campylobacter* revealed by next-generation sequencing in patients with common variable immunodeficiency

**DOI:** 10.3389/fmicb.2026.1750824

**Published:** 2026-04-01

**Authors:** Quentin Jehanne, Anne Conrad, Raphaële Nove Josserand, Nicolas Benech, Paul Marie Schatz, Lucie Bénéjat, Astrid Ducournau, Johanna Aptel, Moeava Martin, Marine Jauvain, Olivier Dauwalder, Philippe Lehours

**Affiliations:** 1 CHU de Bordeaux, CNR des Campylobacters et des Hélicobacters, Bordeaux, France; 2 Hospices Civils De Lyon, Service Des Maladies Infectieuses Et Tropicales, Lyon, France; 3 Hospices Civils De Lyon, Service De Médecine Interne Et Pathologie Vasculaire, Lyon, France; 4 Hospices Civils De Lyon, Service D’Hépatogastroentérologie, Lyon, France; 5 INSERM U1312, UMR BRIC-Team 4, Université de Bordeaux, Bordeaux, France; 6 Hospices Civils De Lyon, Centre de Biologie et de Pathologie Nord, Institut des Agents Infectieux, Plateau de Microbiologie 24/24, Lyon, France

**Keywords:** bacteremia, *Campylobacter*, carbapenem resistance, immunodeficiency, next-generation sequencing

## Abstract

Because few studies have focused on recurrent *Campylobacter* bacteremia, we investigated two clinical cases of patients with common variable immunodeficiency and repeated *Campylobacter* bacteremia over a period of 6–10 years. We analyzed and compared genomes from isolates obtained from both patients during follow-up. For patient #1, 18 isolates of *Campylobacter coli* and 17 isolates of *Campylobacter jejuni* were obtained from 2014 to 2024. For patient #2, 10 isolates of *C. coli* were obtained from 2019 to 2024. Next-generation sequencing was used to identify species, characterize antimicrobial resistance, perform multilocus sequence typing, and analyze core-genome single-nucleotide polymorphisms, as well as to uncover potential sources of contamination. For patient #1, all 18 *C. coli* isolates obtained from 2022 to 2024 were from the same clonal complex and source of contamination (chicken) and exhibited high levels of genomic resemblance based on core-genome single-nucleotide polymorphism analysis. Each *C. coli* isolate probably originated from the same initial strain. However, two clusters of *C. jejuni* were identified: one consisting of isolates from 2014 and the other consisting of the remaining isolates from 2022 to 2024. A *16S rRNA* mutation in position A1387G was present in four *C. coli* isolates from 2022 and 2023, and this was associated with gentamicin resistance. One *C. coli* isolate was also resistant to ertapenem and exhibited an amino acid duplication within the PorA protein sequence. For patient #2, each *C. coli* isolate was from the same clonal complex, which was of porcine origin. Similar to patient #1, three of the isolates from 2023 had an A1464G *16S rRNA* mutation and were gentamicin resistant. Retrospective analyses of antimicrobial use for both patients highlighted an association between antimicrobial selection pressure and the emergence of resistance markers, suggesting *in vivo* selection.

## Introduction

*Campylobacter* species, mainly *Campylobacter jejuni* and *Campylobacter coli*, are responsible for the highest number of bacterial gastroenteritis cases worldwide, more than those caused by *Salmonella* species (Chlebicz and Śliżewska, 2018; European Food Safety Authority and European Centre for Disease Prevention and Control, 2025). In 2023, a total of 148,181 cases of campylobacteriosis were reported in Europe, whereas there were only 77,486 cases of salmonellosis (Authority (EFSA) EFS and European Centre for Disease Prevention and Control, 2024). However, mortality rates remain low. In fact, symptoms induced by campylobacteriosis are generally mild; these include abdominal cramps, diarrhea, and fever but healthy individuals usually recover spontaneously (Kaakoush et al., 2015). Nevertheless, rare complications may occur in patients with concomitant diseases such as immunosuppression, diabetes, or cancer, and intestinal infections progress to bacteremia in approximately 1% of cases (Fernández-Cruz et al., 2010; Tinévez et al., 2022).

Immune system disorders are major risk factors for chronic *Campylobacter* infections, even without any gastrointestinal symptoms. This is especially true for primary immunodeficiency diseases such as common variable immunodeficiency (CVID) or, more frequently, X-linked agammaglobulinemia (XLA). In fact, a recent study in the United Kingdom showed that in patients with primary immunodeficiency and chronic or recurrent *Campylobacter* infections, strains can persist for years in the intestinal tract and reinfect the patient (Grammatikos et al., 2023). CVID is characterized by a deficiency in immunoglobulin production and is the leading cause of primary immunodeficiency, with a prevalence of approximately 1 case per 30,000 adults worldwide. CVID includes a range of conditions defined by distinctive genetic and immunological characteristics (Warnatz et al., 2002; Oksenhendler et al., 2008). XLA is a primary immunodeficiency caused by a mutation in the Bruton tyrosine kinase gene and characterized by a deficiency of circulating B lymphocytes and immunoglobulins (Conley and Cooper, 1998). It may be the most common disease associated with recurrent *Campylobacter* infections and affects individuals across a wide range of age groups (Grammatikos et al., 2023).

Relapses of campylobacteriosis are rare in immunocompetent individuals (García-Sánchez et al., 2024). Therefore, it is advisable to look for evidence of immunodepression in cases of recurrent *Campylobacter* infections (Gharamti et al., 2020). These infections can sometimes be complicated by reactive arthritis (Arai et al., 2007), deep-seated infections such as osteomyelitis (Hartman et al., 2020), or cellulitis (Tokuda et al., 2004). The characteristics of both the patient and the strain may strongly influence the duration of infections, and antimicrobial administration combined with immunoglobulin replacement therapy is often necessary to eradicate the problem (Najjar et al., 2020). Although immunoglobulin replacement therapy can counteract *Campylobacter* infections, it does not appear to influence the chronic asymptomatic presence of the bacteria. IgA deficiency may be a risk factor for campylobacteriosis in individuals with CVID or XLA (Oksenhendler et al., 2008; Dion et al., 2019; Roa-Bautista et al., 2023), but some data have highlighted other potential risk factors (Dion et al., 2019; Grammatikos et al., 2023; Markocsy et al., 2024). IgM deficiency may reduce the clearance of *Campylobacter* from the blood, but can also alter intestinal permeability and thus contribute to an increased risk of bacteremia (Merrick et al., 2022). Indeed, standard preparations of polyvalent immunoglobulins contain only very small amounts of IgA and IgM, which may explain recurrent campylobacteriosis even in patients receiving immunoglobulin replacement therapy.

In France (Lehours et al., 2023) and throughout the European Union, *Campylobacter* resistance rates to ciprofloxacin and tetracycline had reached alarming levels by 2023 in human isolates (Authority EFS, European Centre for Disease Prevention and Control, 2025). For *C. jejuni* and *C. coli* species, 71.9 and 75% resistance to ciprofloxacin, respectively, was observed in the European Union, compared to 64.8 and 66.2% in France. Resistance to ciprofloxacin is associated with mutations at position 86 or 90 in the Gyrase A protein (Tang et al., 2017). In total, 47.9% of *C. jejuni* isolates and 68.2% of *C. coli* isolates were resistant to tetracycline in the European Union, compared to 44.1 and 78.9% in France; the main resistance mechanism involved was *tet*(O) or sometimes *tet*(O-32-O) (Sougakoff et al., 1987). In patients with primary immunodeficiency, resistance to antimicrobials such as carbapenems and macrolides may drastically reduce the impact of antimicrobial therapy alone (Ongen et al., 2023; van der Meer et al., 1986; van den Bruele et al., 2010; Zhuo et al., 2024), sometimes proving fatal (Merrick et al., 2022).

In the present study, we describe the clinical and microbiological characteristics of two patients with CVID and recurrent *Campylobacter* infections. A total of 45 isolates were obtained from the two patients and sent to the French National Reference Center for Campylobacters and Helicobacters (NRCCH) for analysis. These included 18 isolates of *C. coli* and 17 isolates of *C. jejuni* from the first patient and 10 isolates of *C. coli* from the second patient. Whole-genome sequencing (WGS) was used to evaluate the adaptation of these strains to antimicrobial therapies. The study highlighted strong selective pressure among the *Campylobacter* populations obtained from these patients and noted the emergence of highly resistant bacterial strains with rare or novel genomic resistance mechanisms.

## Materials and methods

### Clinical cases descriptions

This study describes recurrent infections of *C. jejuni* and *C. coli* in two female patients with CVID from the University Hospital of Lyon (Hospices Civils de Lyon, Lyon, France). Patient #1 was born in 1954 and, in the 1990s, after multiple episodes of otitis media, the appearance of hypogammaglobinemia, and bronchial dilatation, she was diagnosed with CVID. Immunoglobulin (Ig) replacement therapy consisted of Gammagard (Takeda Pharmaceuticals, Cambridge, MA, United States) due to suspected hypersensitivity to IgA. Other relevant comorbidities were as follows: villous atrophy leading to chronic malnutrition; inflammatory-bowel-disease-like enteritis and colitis (treated with adalimumab from June to July 2020 and with ustekinumab from June 2023 to July 2024); and regenerative nodular hyperplasia of the liver, which later progressed to cirrhosis. Additionally, in 2014 she was diagnosed with a gastric adenocarcinoma, which was treated by partial gastrectomy.

From 2014 onward, patient #1 (born in 1954) experienced over 40 episodes of *C. jejuni* or *C. coli* bacteremia despite receiving polyvalent immunoglobulin replacement therapy with IgG trough levels ranging from 9 to 11 g/L. These episodes were either clinically asymptomatic or only associated with mild symptoms (e.g., cellulitis of the limbs, diarrhea, or occasional fevers) and inflammatory biologic syndrome. The bacteremia episodes were initially several months apart but gradually increased in frequency. In 2024, the first episode of ascites occurred, with fluid that contained *C. jejuni*. Asymptomatic bacteremia episodes were not systematically treated with antibiotics. Symptomatic episodes were treated using antibiotics, including amoxicillin-clavulanic acid, azithromycin, or ertapenem, sometimes in combination with gentamicin. The antibiotics chosen were based on antimicrobial susceptibility testing results and/or ease of administration for outpatient ambulatory therapy. Treatment duration varied from 7 days to 6 weeks. Antibiotic suppressive treatment with azithromycin or oral decontamination capsules did not prevent recurrent *Campylobacter* bacteremia. All bacteremias were monomicrobial.

Patient #2 was born in 1947 and had been monitored for CVID since 2014. Her first episode of *C. coli* bacteremia occurred in 2016. Since her CVID diagnosis, patient #2 had undergone subcutaneous and intravenous substitution therapy to maintain IgG levels at 8–9 g/L. She received no additional treatment during the bacteremia episodes. She had a fractured left shoulder in 2017 (prosthesis inserted), a fractured left femur and humerus in 2020 (gamma nail and prosthesis inserted, respectively), and pain in her right hip with associated swelling since September 2021. Subcutaneous samples from the hip were positive for *C. coli*. Arthritis in the right hip was treated by removal of the gamma nail and two courses of ertapenem followed by imipenem. A fecal microbiota transplant was performed in July 2021 but stools have remained positive for *C. coli* since then. Similar to patient #1, clinical stabilization has occurred many times since 2016 but samples have remained positive for *C. coli*. Symptomatic episodes have been treated with antibiotics, including amoxicillin-clavulanic acid, azithromycin, gentamicin, and carbapenems (i.e., ertapenem and imipenem). As for patient #1 all bacteremias were monomicrobial.

To date, patient #2 still has *C. coli* infections; patient #1 died from pneumonia in December 2024.

### Clinical sampling and antimicrobial susceptibility testing

A total of 35 isolates were obtained from patient #1 between 2014 and 2024, including 18 isolates of *C. coli* (designated the 1C pool of isolates in the present study) and 17 isolates of *C. jejuni* (designated the 1J pool of isolates). A total of 10 isolates of *C. coli* were obtained from patient #2 between 2019 and 2024 (designated the 2C pool of isolates). Each isolate was obtained from blood culture except for isolate 2C03 from patient #2, which was obtained from stools in 2022. All *C. coli* and *C. jejuni* were isolated on Columbia blood agar plates with 5% sheep blood (Thermo Fisher Scientific, Waltham, MA, United States) and incubated at 37 °C, following the Referentiel de Microbiologie Clinique (REMIC V7) published in 2022 by the French Society for Microbiology. A microaerobic atmosphere was maintained (79.7%N_2_, 7.1%CO_2_, 6.1%O_2,_ and 7.1%H_2_) using an Anoxomat microprocessor (Mart Microbiology BV, Lichtenvoorde, the Netherlands). Species identities were confirmed using matrix-assisted laser desorption/ionization time-of-flight mass spectrometry (Bessède et al., 2011). Susceptibility to five routinely monitored antimicrobials (ampicillin, ciprofloxacin, erythromycin, tetracycline, and gentamicin) was assessed using the disk diffusion method, and resistance was also detected using WGS. In addition, minimum inhibitory concentrations (MICs) of three carbapenems (ertapenem, meropenem, and imipenem) were determined using Etest strips (bioMérieux, Marcy-l’Étoile, France). For both analyses, bacterial inoculums at 0.5 McFarland standard were subcultured on Mueller–Hinton agar supplemented with 5% defibrinated horse blood and 20 mg/L nicotinamide adenine dinucleotide (*β*-NAD; bioMérieux). Cultures were incubated for 24–48 h at 36 °C in a microaerobic environment, and data were collected based on the CASFM/EUCAST 2022 guidelines (Comité de l’antibiogramme de la Société Française de Microbiologie, 2022). MICs were measured in mg/L at the point where growth inhibition intersected the strip by two independent readers. For the disk diffusion method, inhibition zone diameters were measured via the SIRscan Auto (i2A, Montpellier, France) automatic system and confirmed manually. The *C. jejuni* ATCC 33560 reference strain was used for quality control.

### WGS and molecular characterizations

WGS of the isolate genomes obtained from patients #1 and #2 were performed using Illumina sequencers (iSeq 100, HiSeq 4,000, NextSeq 500, and NovaSeq 6,000; Illumina, San Diego, CA, United States). Quality control of raw reads and read trimming were performed using FastQC v0.12.0 (Wingett and Andrews, 2018) and Sickle v1.33 (Joshi and Fass, 2011), respectively. Genomes were then assembled using SKESA v2.5.1 (Souvorov et al., 2018). The assembly qualities were checked based on the ECDC recommendations for *Campylobacter* species. Specifically, the assembled genome size should be within the range of 1.5–1.9 Mb, the N50 value should be 30,000 bp (there is no defined threshold), and the total number of contigs should be less than 500, each with a sequence length greater than 300 bp.

Molecular typing of isolates was performed using multilocus sequence typing (MLST) and core genome multilocus sequence typing (cgMLST) methods. Oxford PubMLST schemes (Cody et al., 2017) were downloaded (i.e., allele sequences and profiles), and the Nucleotide-Nucleotide BLAST v2.12.0 + command line tool (Altschul et al., 1997) was used to extract all loci. BLAST was also used to determine molecular antimicrobial resistances, together with various gene and mutation databases (e.g., NCBI, CARD, and ResFinder, as well as the in-house NRCCH *Campylobacter* resistance database). In the context of carbapenem resistance, we performed PorA protein structure homology modeling using the AlphaFold prediction tool (Jumper et al., 2021). Antimicrobial resistance-carrying plasmid sequences were identified using RFPlasmid tools v1.0 (van der Graaf- Bloois et al., 2021). Potential sources of contamination were identified using previously published data for *C. jejuni* and *C. coli*, as well as STRUCTURE software for population genetics inference (Hubisz et al., 2009). Briefly, 15 *C. jejuni* host-segregating markers of the poultry, ruminant and environment reservoirs and 259 *C. coli* SNPs of the poultry, ruminant and pig reservoirs were extracted from each clinical isolate in order to compute a score of attribution (Thépault et al., 2017; Jehanne et al., 2020).

### Core genome comparisons of isolated strains

To obtain an overview of strain adaptation over time, core genomes from 1C, 1J, and 2C isolates were generated, and pairwise comparisons of single-nucleotide polymorphisms (SNPs) were performed. First, each genome was annotated using Prokka v1.14.6 (Seemann, 2014), and pangenomes were generated from annotations outputs using Roary v1.7.8 (Page et al., 2015). Core genomes were constructed from pangenomes by selecting genes shared by all isolates. Raw sequencing data from each isolate from the 1C, 1J, and 2C pool of isolates was aligned against the 1C, 1J, and 2C constructed core genome, respectively, using bwa v0.7.17 (Li, 2013) and SAMtools v1.19.2 (Li, 2011) with default parameters. BCFtools v1.19 (Li, 2011) was used to call variants with the default parameters. The pairwise comparisons were performed for each nucleotide position where a SNP was detected. Distances between each isolate (i.e., the number of different variants between two isolates) were computed using MEGA v11.0.13 (Tamura et al., 2021), and trees were visualized using iTOL v7 (Letunic and Bork, 2024). Finally, EggNOG-mapper v2.1.12 (Cantalapiedra et al., 2021) was used to classify each annotated gene into functional categories and identify biological mechanisms with the most genomic variability. In order to confirm that isolates from the present study originated from the same strains, core genomes were compared with a random selection of 160 additional genomes, namely 10 *C. jejuni* and 10 *C. coli* genomes from each year between 2017 and 2024. Raw reads were aligned against cgMLST Oxford scheme v1 (*n* = 1,343 genes), and neighbor-joining trees of SNP distances were constructed as previously described.

## Results

### Genomic characteristics of isolates

Genomic data from all 45 isolates from both patients were obtained (summarized in Table 1 and complete data in Supplementary Table 1). For the *C. coli* isolates obtained from patient #1 (*n* = 18) and patient #2 (*n* = 10), the genomes had an average size of 1,715,904 bp (minimum genome size of 1,710,634, maximum genome size of 1,718,195 and GC = 30.7%) and 1,772,399 (minimum genome size of 1,761,156, maximum genome size of 1,776,391 and GC = 29.4%), respectively, similar to the *C. coli* reference strain ATCC 51798. The average number of contigs was 33 ± 7 and 61 ± 27, respectively. For patient #1, the total number of coding sequences from Prokka annotations of *C. coli* isolates was 1,775 ± 5, with a core genome of 1,709 genes and an accessory genome of 133 genes; for patient #2, there were 1,833 ± 18 coding sequences from *C. coli* isolates, with a core genome of 1,751 genes and an accessory genome of 146 genes. All *C. coli* isolates from this study were identified as complex clonal 828 strains (CC828) (Table 1). However there were minor changes in sequence types (ST) due to mutations in the *aspA* and *gltA* genes (Supplementary Table 1). In addition, patient #1 *C. coli* isolates were attributed to the poultry reservoir, whereas patient #2 isolates were attributed to the pig reservoir.

**Table 1:** Overview of *Campylobacter coli* and *Campylobacter jejuni* isolates from both patients.

Id	Sampling date	Species	Origin (a)	ST/CC (b)	Source (c)	Molecular antimicrobial resistance detection (d)	Carbapenem susceptibility (MIC in mg/L) (e)
1C01	2022-02-14	*C. coli*	Blood	825/828	Chicken	GyrA-**T86I**; tet(O)	*S*
1C02	2022-04-25	*C. coli*	Blood	825/828	Chicken	GyrA-**T86I**; 23S-**A2075G**; tet(O)	*S*
1C03	2022-05-16	*C. coli*	Blood	825/828	Chicken	GyrA-**T86I**; tet(O)	*S*
1C04	2022-11-25	*C. coli*	Blood	825/828	Chicken	GyrA-**T86I**; tet(O); 16S-**A1387G**	**ERT (1.5)**
1C05	2022-11-25	*C. coli*	Blood	825/828	Chicken	GyrA-**T86I**; tet(O)	**ERT (1.5)**
1C06	2022-12-16	*C. coli*	Blood	825/828	Chicken	GyrA-**T86I**; tet(O); 16S-**A1387G**	**ERT (2)**
1C07	2022-12-16	*C. coli*	Blood	825/828	Chicken	GyrA-**T86I**; tet(O); 16S-**A1387G**	*S*
1C08	2023-01-06	*C. coli*	Blood	825/828	Chicken	GyrA-**T86I**; tet(O); 16S-**A1387G**	*S*
1C09	2023-03-10	*C. coli*	Blood	825/828	Chicken	GyrA-**T86I**; tet(O)	*S*
1C10	2023-04-26	*C. coli*	Blood	825/828	Chicken	GyrA-**T86I**; tet(O)	**ERT (2)**
1C11	2023-11-28	*C. coli*	Blood	14,736/828	Chicken	GyrA-**T86I**; tet(O)	*S*
1C12	2024-01-09	*C. coli*	Blood	14,732/828	Chicken	GyrA-**T86I**; tet(O)	*S*
1C13	2024-01-09	*C. coli*	Blood	14,732/828	Chicken	GyrA-**T86I**; tet(O)	*S*
1C14	2024-01-09	*C. coli*	Blood	14,732/828	Chicken	GyrA-**T86I**; tet(O)	*S*
1C15	2024-03-15	*C. coli*	Blood	14,737/828	Chicken	GyrA-**T86I**; tet(O)	*S*
1C16	2024-03-15	*C. coli*	Blood	14,737/828	Chicken	GyrA-**T86I**; tet(O)	*S*
1C17	2024-09-21	*C. coli*	Blood	825/828	Chicken	GyrA-**T86I**; tet(O)	*S*
1C18	2024-09-21	*C. coli*	Blood	825/828	Chicken	GyrA-**T86I**; tet(O)	*S*
1 J01	2014-03-26	*C. jejuni*	Blood	21/21	Cattle	GyrA-**T86I** + **D90N**	*S*
1 J02	2014-03-26	*C. jejuni*	Blood	12,324/21	Cattle	blaOXA581-**G63T**; GyrA-**T86I** + **D90N**	*S*
1 J03	2022-05-16	*C. jejuni*	Blood	14,728/21	Cattle	*blaOXA193*-**G63T**; GyrA-**T86I**; 23S-**A2075G**; tet(O)_pla	*S*
1 J04	2022-07-07	*C. jejuni*	Blood	14,728/21	Cattle	*blaOXA193*-**G63T**; GyrA-**T86I**; 23S-**A2075G**; tet(O)_pla	*S*
1 J05	2023-03-10	*C. jejuni*	Blood	14,728/21	Cattle	*blaOXA193*-**G63T**; GyrA-**T86I**; 23S-**A2075G**; tet(O)_pla	*S*
1 J06	2023-03-31	*C. jejuni*	Blood	14,728/21	Cattle	*blaOXA193*-**G63T**; GyrA-**T86I**; 23S-**A2075G**; tet(O)_pla	*S*
1 J07	2023-04-26	*C. jejuni*	Blood	14,728/21	Cattle	*blaOXA193*-**G63T**; GyrA-**T86I**; 23S-**A2075G**; tet(O)_pla	*S*
1 J08	2023-11-28	*C. jejuni*	Blood	14,728/21	Cattle	blaOXA193-**G63T**; GyrA-**T86I**; 23S-**A2075G**; tet(O)	*S*
1 J09	2024-01-09	*C. jejuni*	Blood	14,728/21	Cattle	blaOXA193-**G63T**; GyrA-**T86I**; 23S-**A2075G**	*S*
1 J10	2024-01-09	*C. jejuni*	Blood	14,728/21	Cattle	blaOXA193-**G63T**; GyrA-**T86I**; 23S-**A2075G**; tet(O)	*S*
1 J11	2024-01-09	*C. jejuni*	Blood	14,728/21	Cattle	blaOXA193-**G63T**; GyrA-**T86I**; 23S-**A2075G**	*S*
1 J12	2024-02-06	*C. jejuni*	Blood	14,728/21	Cattle	blaOXA193-**G63T**; GyrA-**T86I**; 23S-**A2075G**; tet(O)	*S*
1 J13	2024-02-06	*C. jejuni*	Blood	14,728/21	Cattle	blaOXA193-**G63T**; GyrA-**T86I**; 23S-**A2075G**	*S*
1 J14	2024-03-15	*C. jejuni*	Blood	14,728/21	Cattle	blaOXA193-**G63T**; GyrA-**T86I**; 23S-**A2075G**	*S*
1 J15	2024-04-03	*C. jejuni*	Blood	14,728/21	Cattle	blaOXA193-**G63T**; GyrA-**T86I**; 23S-**A2075G**	*S*
1 J16	2024-07-10	*C. jejuni*	Blood	14,729/21	Cattle	blaOXA193-**G63T**; GyrA-**T86I**; 23S-**A2075G**	*S*
1 J17	2024-09-21	*C. jejuni*	Blood	14,728/21	Cattle	blaOXA193-**G63T**; GyrA-**T86I**; 23S-**A2075G**	*S*
2C01	2019-06-07	*C. coli*	Blood	14,731/828	Pig	blaOXA193-**G63T**; GyrA-**T86I**; 23S-**A2075G**; tet(O)	*S*
2C02	2022-07-06	*C. coli*	Blood	14,731/828	Pig	blaOXA193-**G63T**; GyrA-**T86I**; 23S-**A2075G**; tet(O)	*S*
2C03	2022-10-04	*C. coli*	Stools	14,731/828	Pig	blaOXA193-**G63T**; GyrA-**T86I**; 23S-**A2075G**; tet(O); 16S-**G1464T**	*S*
2C04	2023-01-05	*C. coli*	Blood	14,731/828	Pig	blaOXA193-**G63T**; GyrA-**T86I**; 23S-**A2075G**; tet(O); 16S-**G1464T**	**ERT + MER (>32)**
2C05	2023-02-21	*C. coli*	Blood	14,731/828	Pig	blaOXA193-**G63T**; GyrA-**T86I**; 23S-**A2075G**; tet(O)	*S*
2C06	2023-03-27	*C. coli*	Blood	14,730/828	Pig	blaOXA193-**G63T**; GyrA-**T86I**; 23S-**A2075G**; tet(O); 16S-**G1464T**	*S*
2C07	2024-05-02	*C. coli*	Blood	14,731/828	Pig	blaOXA193-**G63T**; GyrA-**T86I**; 23S-**A2075G**; tet(O)	*S*
2C08	2024-06-04	*C. coli*	Blood	14,731/828	Pig	blaOXA193-**G63T**; GyrA-**T86I**; 23S-**A2075G**; tet(O)	*S*
2C09	2024-07-10	*C. coli*	Blood	14,731/828	Pig	blaOXA193-**G63T**; GyrA-**T86I**; 23S-**A2075G**; tet(O)	*S*
2C10	2024-09-24	*C. coli*	Blood	14,731/828	Pig	blaOXA193-**G63T**; GyrA-**T86I**; 23S-**A2075G**; tet(O)	*S*

The data for all strains and corresponding genomes analyzed are shown. Isolates of *C. coli* (*n* = 18) and *C. jejuni* (*n* = 17) from patient #1 are labeled 1C and 1 J, respectively. Isolates of *C. coli* from patient #2 (*n* = 10) are labeled 2C. Each isolate was obtained from blood culture, with the exception of the 2C03 isolate (a). The following novel sequence types (STs) were submitted (b): ST-14728, ST-14729, ST-14730, ST-14731, ST-14732, and ST-14737. Probabilistic estimates of sources of contamination are also shown (c). Antimicrobial resistance genes and mutations are shown in italics and in bold (d), respectively. Etest results for carbapenems are indicated as susceptible (S) or resistant to ertapenem (ERT) and/or meropenem (MER) (e). Additional data for each isolate are shown in Supplementary Table 1.

The assembled genomes for *C. jejuni* isolates from patient #1 were 1,640,254 bp in size (minimum genome size of 1,638,492, maximum genome size of 1,642,016 and GC = 29.8%) for the isolates from 2014 (*n* = 2) and 1,731,268 bp (minimum genome size of 1,720,754, maximum genome size of 1,742,061 and GC = 31.2%) for the remaining isolates (*n* = 15); the average number of contigs was 33 ± 4 and 52 ± 32, respectively. Gene annotations showed substantial differences between the isolates from 2014 and the recent isolates, with 1,693 and 1, 811 ± 14 coding sequences, respectively. The core and accessory genome without the isolates from 2014 had 1,688 (1,483 if all isolates were included) and 221 genes, respectively. Clustering analyses were performed using the dataset containing the 1,688 genes. Similar to the *C. coli* isolates, MLST analysis showed that the *C. jejuni* isolates were all CC-21 strains, with STs variability due to mutations in the *gltA* gene (Supplementary Table 1). All of these isolates were attributed to the cattle reservoir.

### Core genome clustering

**Figure 1:**
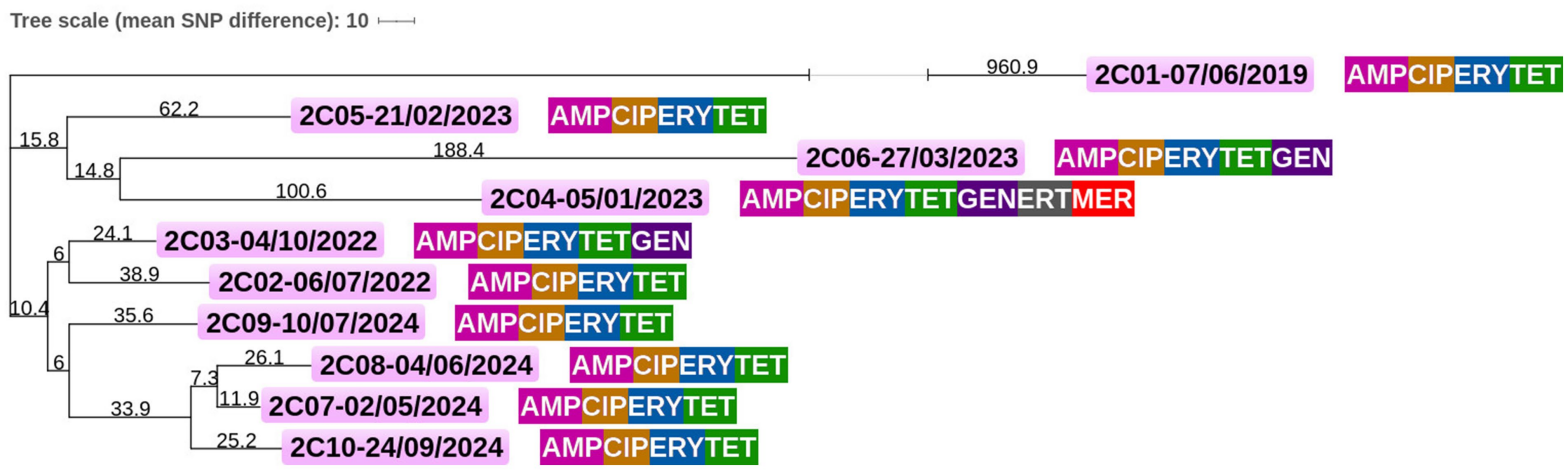
Core genome single-nucleotide polymorphism (cgSNP) tree based on *Campylobacter coli* isolates from patient #1. Raw sequencing data for all 18 isolates of *C. coli* obtained from patient #1 from 2022 to 2024 were aligned against the core genome dataset of 1,709 genes. Pairwise SNP distances were computed by comparing nucleotide differences across the core genome for each isolate. These distances were then used to construct a distance-based phylogenetic tree, where the length of each branch corresponds to the average number of genotype differences between isolates. Antimicrobial resistance for each isolate is indicated as follows: CIP, ciprofloxacin; TET, tetracycline; ERT, ertapenem; GEN, gentamicin; and ERY, erythromycin. Sampling date format is as follows: Day/Month/Year.

**Figure 2:**
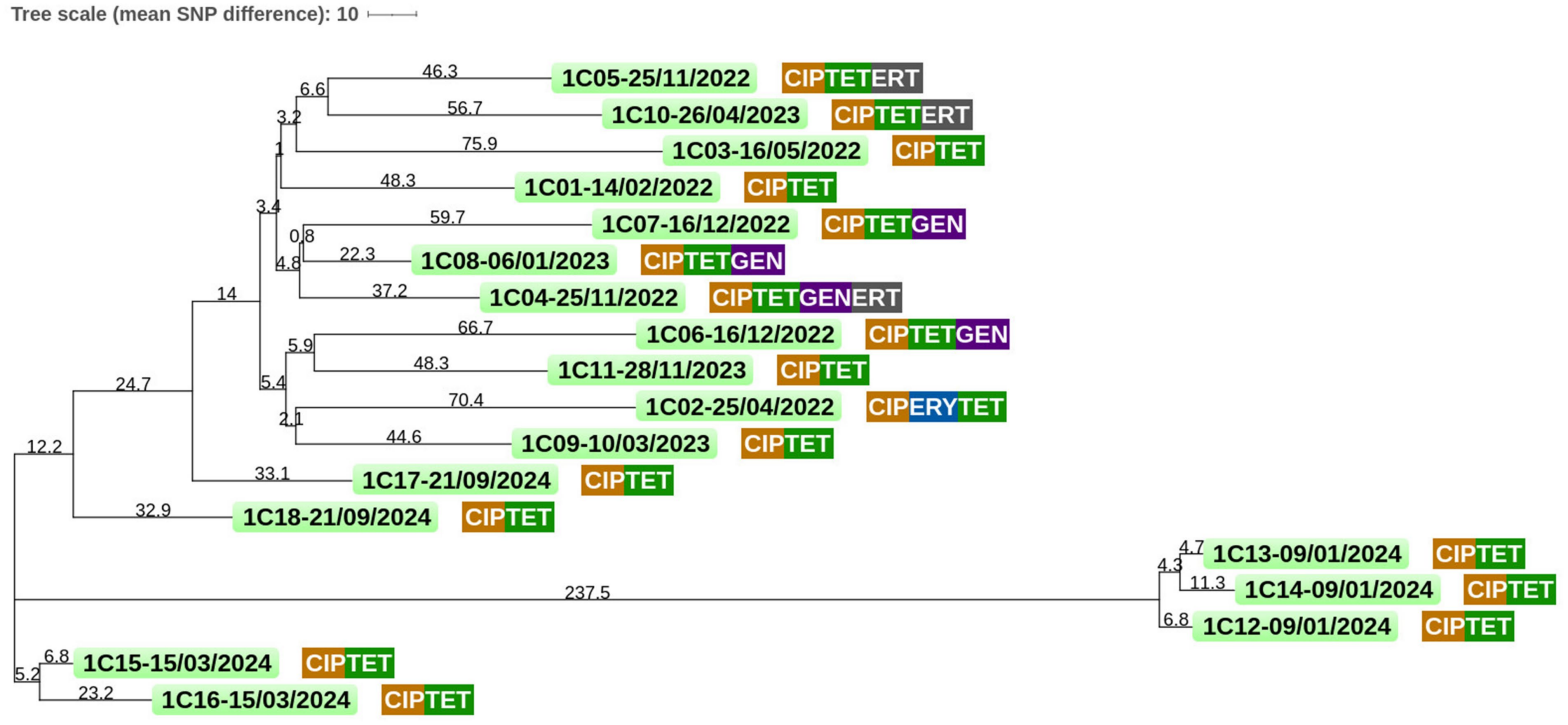
Core genome single-nucleotide polymorphism (cgSNP) tree based on *C. coli* isolates from patient #2. Raw sequencing data for all 10 isolates of *C. coli* obtained from patient #2 from 2019 to 2024 were aligned against the core genome dataset of 1,751 genes. Pairwise SNP distances were computed by comparing nucleotide differences across the core genome for each isolate. These distances were then used to construct a distance-based phylogenetic tree, where the length of each branch corresponds to the average number of genotype differences between isolates. Antimicrobial resistance for each isolate is indicated as follows: AMP, ampicillin; CIP, ciprofloxacin; ERY, erythromycin; TET, tetracycline; GEN, gentamicin; ERT, ertapenem; and MER, meropenem. Sampling date format is as follows: Day/Month/Year.

**Figure 3:**
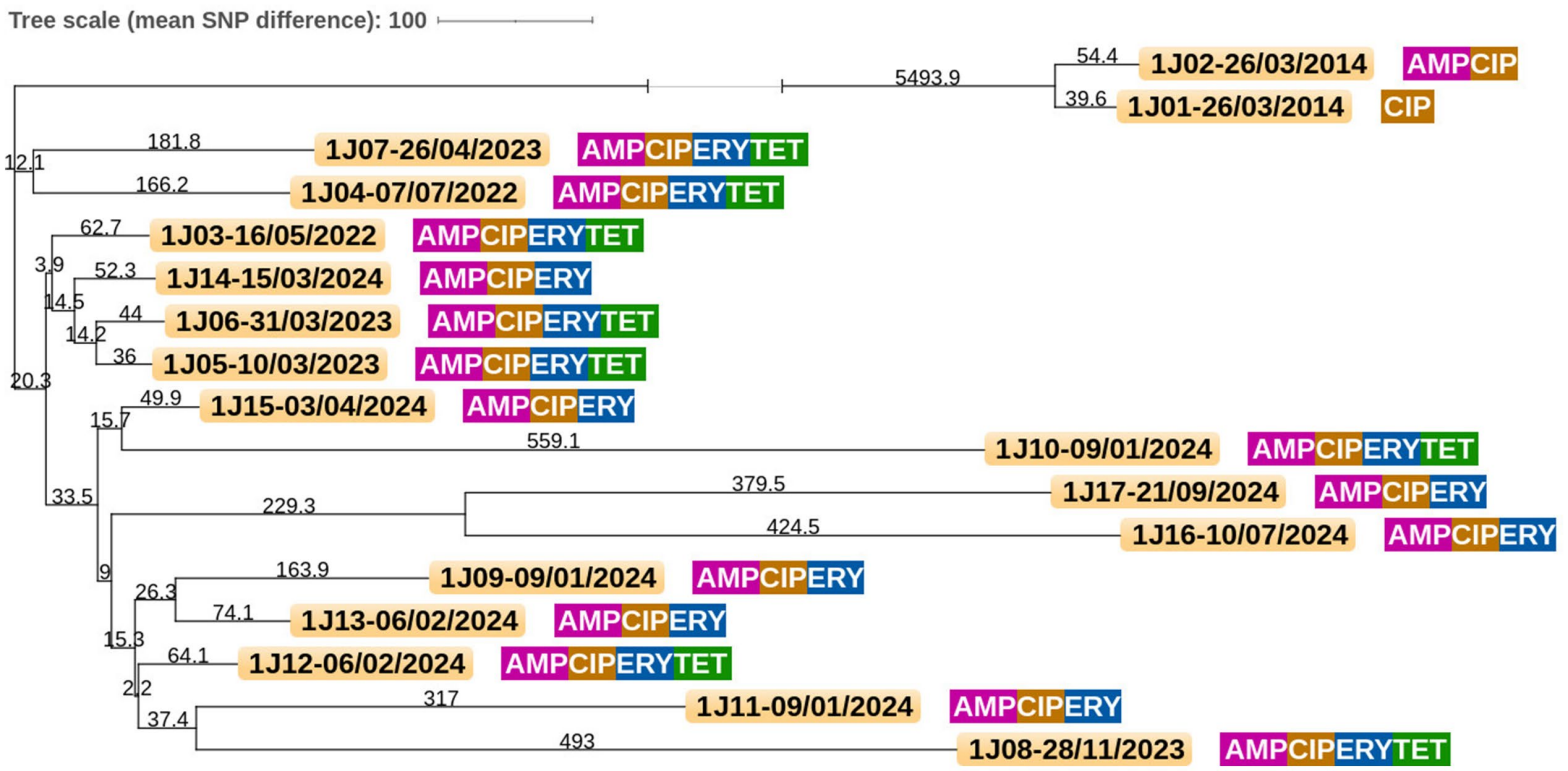
Core genome single-nucleotide polymorphism (cgSNP) tree based on *Campylobacter jejuni* isolates from patient #1. Raw sequencing data for all 17 isolates of *C. jejuni* obtained from patient #1 from 2014 to 2024 were aligned against the core genome dataset of 1,688 genes. Pairwise SNP distances were computed by comparing nucleotide differences across the core genome for each isolate. These distances were then used to construct a distance-based phylogenetic tree, where the length of each branch corresponds to the average number of genotype differences between isolates. Antimicrobial resistance for each isolate is indicated as follows: AMP, ampicillin; CIP, ciprofloxacin; ERY, erythromycin; and TET, tetracycline. Sampling date format is as follows: Day/Month/Year.

**Figure 4:**
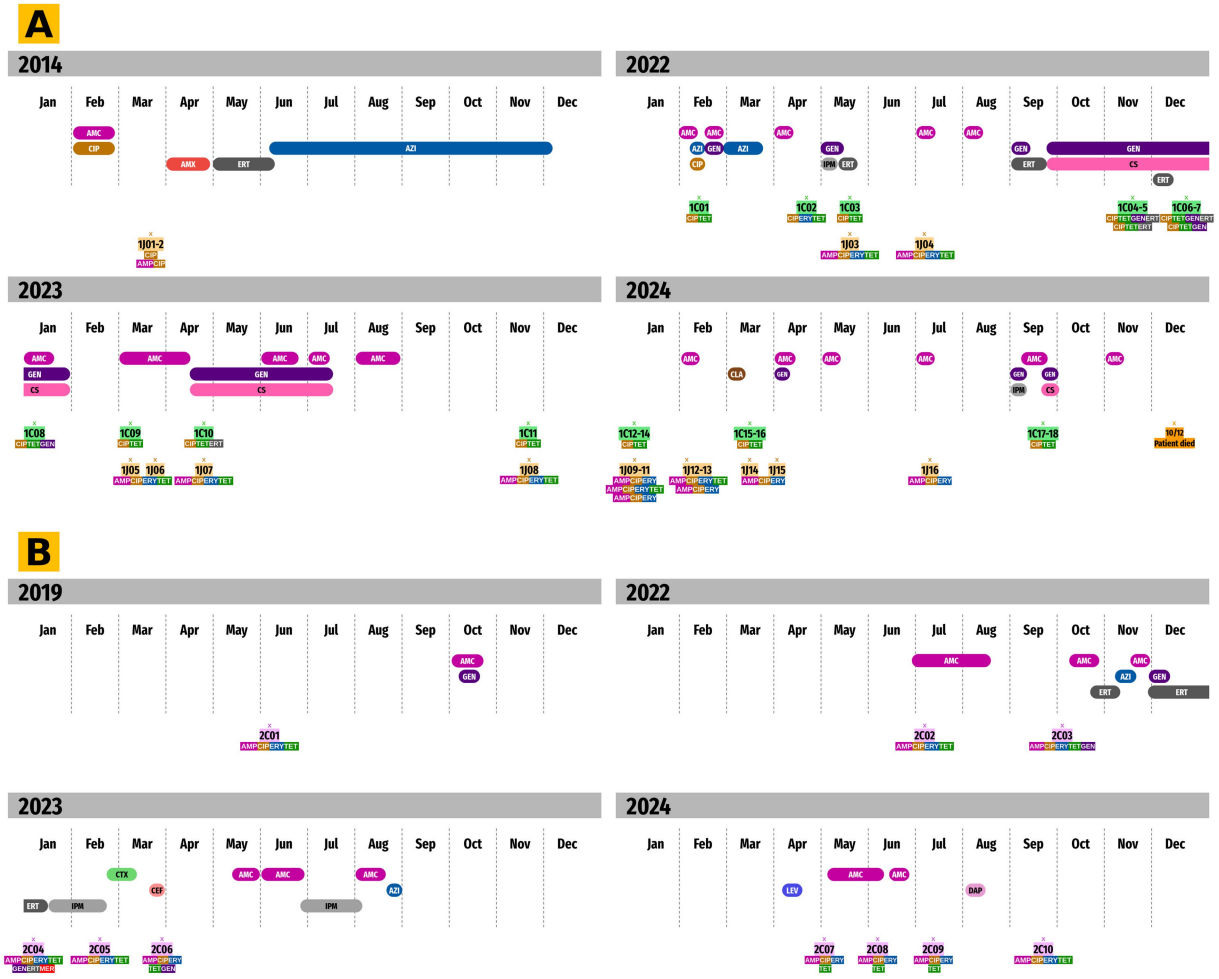
Antimicrobial prescription timelines for patients #1 and #2. The prescription timelines for antimicrobials administered to patient #1 from 2014 to 2024 **(A)** and to patient #2 from 2019 to 2024 **(B)** are shown. The first three rows list antimicrobial prescriptions and their durations. The final two rows list *C. coli* (highlighted in green and purple) and *C. jejuni* (highlighted in orange) isolates along with their resistance profiles. Missing years are not shown because no isolates were sequenced (complete prescriptions data is available in Supplementary Table 4). Antimicrobial prescriptions and resistance are indicated as follows: AMC, amoxicillin-clavulanic acid; GEN, gentamicin; ERT, ertapenem; AZI, azithromycin; IPM, imipenem; CTX, ceftriaxone; CEF, cefepime; LEV, levofloxacin; DAP, daptomycin (*Staphylococcus hominis* infection); CIP, ciprofloxacin; AMX, amoxicillin; CS, colistin; and CLA, clarithromycin.

Analyses using core genome data revealed strong similarities among isolates from the present study. *C. coli* isolates from patient #1 from 2022 to 2024 were on average 183 SNPs distant from each other, using the core genome dataset generated from the Prokka annotations (the closest isolates, 1C13 and 1C14, were distant from only 16 SNPs and the most distant isolates, 1C14 and 1C03, from 388 SNPs) (Figure 1). *C. coli* isolates from patient #2 also revealed strong similarities with low mutation rates (the closest isolates, 2C07 and 2C08, were distant from 38 SNPs and the most distant isolates, 2C01 and 2C06, from 1,180 SNPs) (Figure 2). The corresponding genomes from 2019 to 2022 were on average 337 SNPs distant from each other, and only 158 SNPs apart if the most distant *C. coli* isolate from 2019 (2C01) was excluded. Higher mutation rates were exhibited by *C. jejuni* isolates from patient #1 (the closest isolates, 1 J05 and 1 J06, were distant from 80 SNPs and the most distant isolates, 1 J10 and 1 J16, from 1,238 SNPs) (Figure 3). On average, the corresponding genomes from 2022 to 2024 (*n* = 15) were 534 SNPs apart, indicating a close relationship between these isolates. However, when 1 J01 and 1 J02 were included (i.e., considering all isolates from 2014 to 2024, *n* = 17), the average number of SNPs increased to 1,700. These two isolates from 2014 were an average of 5,541 SNPs distant from the remaining 15 isolates and may be originated from a relatively distant strain. cgMLST analysis using Oxford scheme v1 and 160 additional genomes supported the core genome clustering results based on the Prokka annotations (Figure 4, data of additional genomes in Supplementary Table 2). *Campylobacter coli* isolates from patients #1 and #2 formed distinct clusters (Figure 4A), and *C. jejuni* isolates from patient #1 separated into two groups (Figure 4B): isolates from 2014 (1 J01 and 1 J02) and the remaining isolates from 2022 to 2024. However, there were no clear associations between the genome clustering data and antimicrobial resistance or sampling dates, except for 1C isolates 11–18 from late 2023 to 2024. Those particular isolates were resistant to both ciprofloxacin and tetracycline; they were apparently distinct from the other *C. coli* isolates from this patient.

### Core genome comparisons of isolated strains

Functional categories of core genome genes of isolates from both patients were predicted, and are shown in Table 2 and Supplementary Table 3. Overall, most SNPs observed for the two cases included in our study were in genes associated with bacterial adaptations and had functions such as defense, energy metabolism (e.g., amino acids, lipids, and carbohydrates), and motility. In particular, *C. coli* isolates from patient #1 had two defense genes with many mutations: efflux pump membrane transporter *bepE* with 35 SNPs and a putative glycosyl transferase with 31 SNPs. Genes with many mutations from *C. coli* isolates from patient #2 were associated with amino acid, nucleotide, and lipid transport and metabolism, such as *metC* with 120 SNPs, aspartate ammonia-lyase *aspA* with 84 SNPs, and *ispG* with 89 SNPs. Many SNPs were also present in energy-related genes such as catalase *katA* (78 SNPs), C4-dicarboxylate *dcuD* (59 SNPs), and a putative oxidoreductase (44 SNPs). In addition, *C. jejuni* isolates from patient #1 exhibited 160 SNPs in *aspA* and 133 SNPs in the metabolism-related gene *purB* (an adenylosuccinate lyase subfamily gene).

**Table 2:** Variant calling of genes from the core genome of each species and patient.

Categories (a)	Patient #1—*C. jejuni*			Categories (a)	Patient #1—*C. coli*			Categories (a)	Patient #2—*C. coli*		
	SNPs (b)	Genes (c)	SNPs/gene (d)		SNPs (b)	Genes (c)	SNPs/gene (d)		SNPs (b)	Genes (c)	SNPs/gene (d)
Amino acid	485	17	28.5	Defense	67	3	22.3	Lipid	170	8	21.3
Nucleotide	223	11	20.3	Motility	74	12	6.2	Energy	261	14	18.6
Posttranslational	244	13	18.8	Energy	130	22	5.9	Amino acid	328	20	16.4
Cell cycle	72	4	18.0	Carbohydrate	40	8	5.0	Carbohydrate	86	6	14.3
Defense	65	5	13.0	Amino acid	71	15	4.7	Signal	75	8	9.4
Translation	180	16	11.3	Signal	65	14	4.6	Motility	99	12	8.3
Trafficking	104	10	10.4	Coenzyme	67	17	3.9	Ion transport	73	13	5.6
Motility	125	13	9.6	Transcription	31	12	2.6	Unknown	173	46	3.8
Energy	76	8	9.5	Posttranslational	49	20	2.5	Membrane	59	18	3.3
Coenzyme	85	9	9.4	Unknown	161	84	1.9	Transcription	32	10	3.2
Ion transport	185	23	8.0	Ion transport	31	19	1.6	Secondary metabolites	15	5	3.0
Unknown	399	53	7.5	Lipid	13	9	1.4	Recombination	24	8	3.0
Signal	72	10	7.2	Translation	17	14	1.2	Nucleotide	12	5	2.4
Transcription	41	9	4.6	Membrane	29	26	1.1	Translation	22	12	1.8
Recombination	29	7	4.1	Recombination	12	12	1.0				
Carbohydrate	11	4	2.8								
Membrane	46	19	2.4								

Each gene was classified into functional categories (a) based on EggNOG-mapper annotation. For each category, the total number of single-nucleotide polymorphisms (b) and genes (c) resulting from the alignment of isolate raw sequencing data and the core genomes are shown. Results are sorted by categories according to the number of mutations per gene (d). The EggNOG-mapper category labels are as follows: Amino Acid = Amino acid transport and metabolism; Carbohydrate = Carbohydrate transport and metabolism; Cell Cycle = Cell cycle control, cell division, and chromosome partitioning; Coenzyme = Coenzyme transport and metabolism; Defense = Defense mechanisms; Energy = Energy production and conversion; Ion Transport = Inorganic ion transport and metabolism; Lipid = Lipid transport and metabolism; Membrane = Cell wall/membrane/envelope biogenesis; Motility = Cell motility; Nucleotide = Nucleotide transport and metabolism; Posttranslational = Posttranslational modification, protein turnover, and chaperones; Recombination = Replication, recombination, and repair; RNA = RNA processing and modification; Secondary Metabolites = Secondary metabolites biosynthesis, transport, and catabolism; Signal = Signal transduction mechanisms; Trafficking = Intracellular trafficking, secretion, and vesicular transport; Transcription = Transcription; Translation = Translation, ribosomal structure, and biogenesis; Unknown = Function unknown.

### Antimicrobial resistance genes and mutations

Resistance to the following seven antimicrobials was observed using disk diffusion or Etest and computer-based analyses: ampicillin, ciprofloxacin, erythromycin, tetracycline, gentamicin, ertapenem, and meropenem (Table 1). We noted five previously described resistance mechanisms as well as two potentially novel ones. A point mutation G57T in the promoter region of the *blaOXA* gene (*blaOXA*-581 in 1 J02 and *blaOXA*-193 in 1 J03 to 1 J17 and 2C01 to 2C10) was associated with ampicillin resistance (Zeng et al., 2014) and was present in all *C. jejuni* isolates from patient #1 (except 1 J01) and all *C. coli* isolates from patient #2. All *C. coli* isolates from patient #1 were susceptible to ampicillin. The mutation T86I in the GyrA protein (combined with the D90N mutation for isolates 1 J01 and 1 J02) was associated with ciprofloxacin resistance and was found in all 45 *Campylobacter* isolates (Engberg et al., 2001). Resistance to erythromycin was associated with the *23S rRNA* gene mutation A2075G (Engberg et al., 2001), and was found in all *C. jejuni* isolates from patient #1 obtained after 2014 and all *C. coli* isolates from patient #2. Only one *C. coli* isolate from patient #1 from 2022 (1C02) also displayed the A2075G mutation. Every *C. coli* isolate from this study, both from patient #1 and #2, was resistant to tetracycline due to the presence of a chromosomal *tet*(O) gene (Sougakoff et al., 1987). On the other hand, *C. jejuni* isolates were resistant to tetracycline when they had either chromosomal or plasmid *tet*(O), which included the 1 J isolates from May 16th of 2022 to April 26th of 2023. Four isolates of *C. coli* from patient #1 (1C04, 1C06, 1C07, and 1C08) had a A1387G mutation in the *16S rRNA* gene, which is associated with gentamicin resistance (Zarske et al., 2024). We also found a novel G1464T mutation in the *16S rRNA* of three *C. coli* isolates from patient #2 that were resistant to gentamicin (2C03, 2C04, and 2C06). Finally, *C. coli* isolate 2C04 exhibited a DNAIDGL motif duplication in the PorA protein in position 139 and may be associated with high MICs of ertapenem and meropenem (>32 mg/L). Interestingly, protein structure homology modeling of this sequence using AlphaFold protein structure prediction (Jumper et al., 2021) showed an obstruction in the PorA ion channel (Supplementary Figure 1).

### Antimicrobial selective pressure

**Figure 5:**
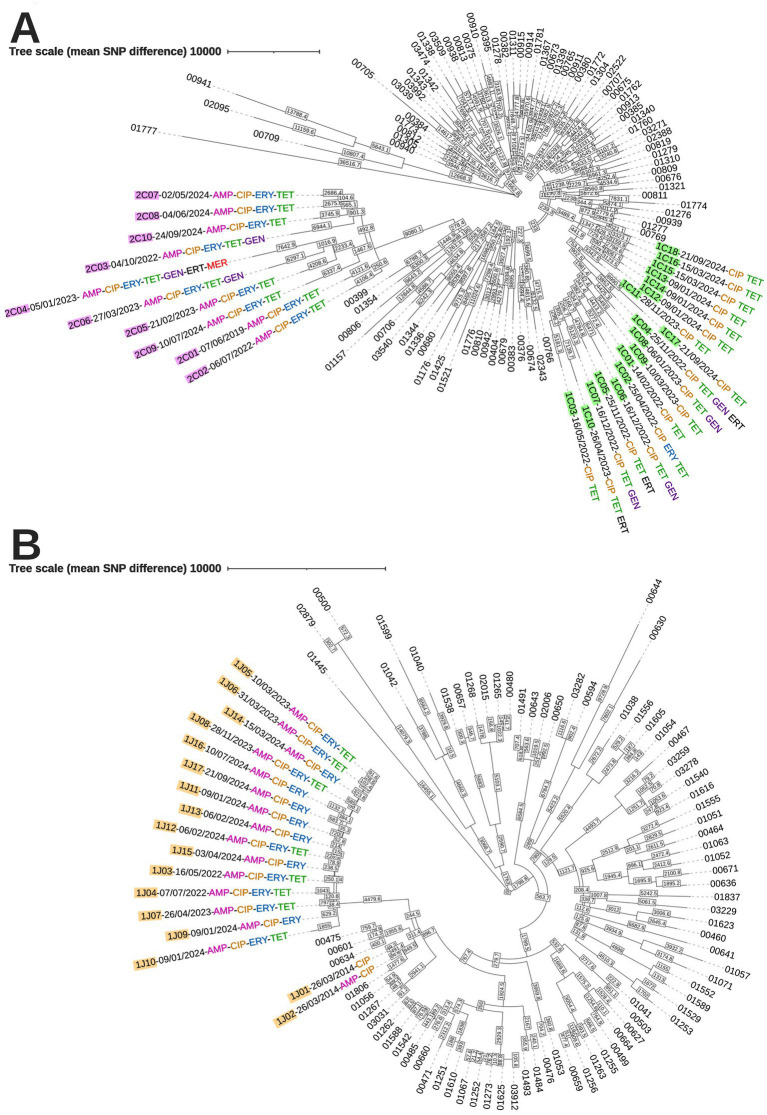
Pairwise SNP comparisons of studied isolates and 160 additional *C. jejuni* and *C. coli* genomes using core genome multilocus sequence typing (cgMLST). Isolates from both patients #1 and #2 (*n* = 45) and 160 randomly selected genomes from the National Reference Center for Campylobacters and Helicobacters (80 *C. jejuni* and 80 *C. coli* genomes obtained from 2017 to 2024) were aligned against 1,343 genes from cgMLST Oxford scheme v1. Trees were constructed from pairwise SNP differences between isolates. The tree on the top shows the *C. coli* isolates **(A)**, whereas the tree on the bottom shows the *C. jejuni* isolates **(B)**. *C. coli* and *C. jejuni* isolates from patient #1 are highlighted in green and orange, respectively, whereas *C. coli* isolates from patient #2 are highlighted in purple. Antimicrobial resistance for each isolate is indicated as follows: AMP, ampicillin; CIP, ciprofloxacin; ERY, erythromycin; TET, tetracycline; ERT, ertapenem; GEN, gentamicin; and MER, meropenem. Sampling date format is as follows: Day/Month/Year. Data for the additional 160 isolates are shown in Supplementary Table 2.

We observed associations between antimicrobial prescriptions and the emergence of resistance among *C. coli* and *C. jejuni* isolates from both patients. For patient #1, although ampicillin, ciprofloxacin, and tetracycline resistance markers were consistently found among both species isolates over time regardless of antimicrobial use, three notable events were identified (Figure 5A). After a period of azithromycin exposure between mid-February and mid-March 2022, erythromycin resistance was detected within 1C02 (sampled in April) and 1 J03 (sampled in May) isolates through the A2075G mutation in the *23S rRNA* sequence. This mutation persisted among the *C. jejuni* isolates but not the *C. coli* isolates. Similarly, an A1387G mutation emerged in the *16S rRNA* sequence among *C. coli* isolates (1C04, 1C06, 1C07, and 1C08) (Figure 5A) after a long period of intravenous gentamicin followed by oral gentamicin exposure from September 2022 to January 2023. However, no samples were collected between March and May 2023, after the oral gentamicin prescription. Finally, ertapenem resistance was observed in 1C04, 1C05, and 1C06 *C. coli* isolates in 2022, possibly as a result of ertapenem prescription in September and December of the same year. For *C. coli* isolates from patient #2, antimicrobial selective pressure due to prescriptions was less noticeable (Figure 5B). Every isolate that was obtained from 2019 to 2024 was resistant to ampicillin, ciprofloxacin, erythromycin, and tetracycline. Resistance to gentamicin emerged in October 2022, as well as in January and March 2023, but with no link to any aminoglycoside prescription. However, after a long period of ertapenem exposure between December 2022 and January 2023, an isolate that was highly resistant to carbapenems (2C04) was obtained, with MICs above 32 mg/L. This resistance is probably associated with the DNAIDGL motif duplication in the PorA protein. The complete antimicrobial prescriptions dataset is shown in Supplementary Table 4.

## Discussion

In this study, we describe recurrent bacteremia infections occurring over a period of 6 and 10 years in two patients with CVID at Lyon University Hospital. Phenotypic and next-generation sequencing analyses were used to characterize the strains, demonstrate recurrence, show how the same strain had adjusted its resistance profile in response to different antibiotic treatments, and identify genetic variations that may reflect *in vivo* adaptations.

In primary immunodeficiency diseases such as CVID and XLA, the long-term presence of *Campylobacter* in the gut may be complicated by bacteremia (Dionisi et al., 2011). The prevalence of *Campylobacter* in the gut of these patients remains unclear, although there have been a few retrospective studies that include patients with CVID (Cunningham-Rundles and Bodian, 1999; Dion et al., 2019; Hartman et al., 2020; Roa-Bautista et al., 2023), and prevalence may vary from 1 to 20% (Dion et al., 2019; Dionisi et al., 2011; Ariganello et al., 2013). This variation is due to the diversity of patients included in the studies and the different approaches used to diagnose campylobacteriosis. Isolation by stool culture is less sensitive than diagnosis by polymerase chain reaction (PCR).

A few studies have investigated the evolution of *Campylobacter* strains responsible for chronic infections in immunocompromised patients (Moore et al., 2001; Barker et al., 2020). As an example, Barker et al. studied 25 *C. jejuni* isolates over a 15-year period using WGS. This raises a legitimate question. Are patients infected by evolving strains, or by virtually identical strains multiple times? Our bioinformatic analyses support the former hypothesis. Barker et al. (2020) demonstrated the persistence of a unique strain while highlighting genetic microevolution and the gradual emergence of macrolide-resistant markers during antibiotic therapy. The possibilities for *Campylobacter* spp. contamination in humans are diverse (Chlebicz and Śliżewska, 2018). It is highly unlikely that the same clones could have repeatedly infected our patients over a period of 6 to 10 years.

Most of the loci identified by Barker et al. encoded surface proteins, suggesting possible links to the host immune system. Other studies have found that against a background of chronic infections, gradual changes may occur in genes associated with motility and chemotaxis (Dunn et al., 2018). Our results are consistent with these earlier studies. Indeed, many of the SNPs observed in the two cases included in our study were in genes associated with defense, energy metabolism, and motility.

Other factors may also explain strain variability over long periods of infection, such as multiple antibiotic treatments, immunotherapy, gut microbiota, co-infections, or any changes that may be associated locally with nutrient loss (Fadlallah et al., 2018; Ling et al., 2025). Differences in microbiota diversity in patients with CVID compared with healthy patients have been described (Bosák et al., 2021). In addition, the quantity and diversity of antibiotic treatments administered to these patients in an attempt to eliminate all digestive carriage, or in the context of recurrent bacteremia, not only contribute to dysbiosis but also to the selection of mutants resistant to these treatments. The efficacy of antibiotic for these chronic infections in patients with CVID has been evaluated in previous studies (Arai et al., 2007; Hartman et al., 2020; Roa-Bautista et al., 2023; Zhuo et al., 2024; Nunes et al., 2023; Okada et al., 2008; Díaz-Alberola et al., 2024). Roa-Bautista et al. obtained intestinal bacterial clearance with long-term treatment (10 days to 7 weeks) using various combinations of azithromycin, chloramphenicol, tigecycline, ertapenem, and aminoglycosides (e.g., gentamicin, tobramycin, or neomycin) (Roa-Bautista et al., 2023). Apparently, oral aminoglycosides can eliminate digestive *Campylobacter* carriage in these patients. Some studies have used neomycin (Nunes et al., 2023) or kanamycin (Okada et al., 2008). In patient #1, however, prolonged oral intake of decontamination capsules of gentamicin led to the emergence of a strain resistant to gentamicin. Carbapenems have also been combined with gentamicin in other studies (Hartman et al., 2020; Díaz-Alberola et al., 2024). Some authors have suggested combining carbapenems with oral tetracycline to eliminate the digestive reservoir of *Campylobacter* (Arai et al., 2007; Zhuo et al., 2024), but this was not an option for the two cases described in our study because the strains were resistant to tetracyclines *in vitro*.

Okada et al. found that five of the six cases of chronic infection that they described had no gastrointestinal symptoms or diarrhea (Okada et al., 2008). Ariganello et al. confirmed the persistence of *C. jejuni* carriage in a child with XLA; there were no digestive symptoms but there were recurring episodes of bacteremia (Ariganello et al., 2013). The various resistance profiles of strains isolated from blood may reflect the translocation of a main clone within a digestive flora consisting of variants whose proportions are influenced by antibiotic therapy and the host response. The causes of chronic digestive carriage in immunocompromised patients are complex and probably linked to a defective humoral response. Tinevez et al. showed that *Campylobacter* bacteremia in immunocompromised patients was associated with an increased risk of mortality and secondary localization (Tinévez et al., 2022). Surprisingly, for the two patients described in our study, most of these episodes were well tolerated. However, in October 2022 patient #2 had arthritis in the right hip due to *Campylobacter*, which was treated by removal of the gamma nail and two prolonged courses of ertapenem followed by imipenem. Therefore, further studies are needed to understand the relevant immunological mechanisms associated with digestive translocation and bacteremia episodes.

The strains identified in our study are highly subject to mutation. In both patients, a mutation appeared in the *16S rRNA* sequence (A1387G or G1464T) and was potentially associated with gentamicin resistance (Zarske et al., 2024). This mechanism has also been described in *Mycobacterium abscessus* (Nessar et al., 2011) but is unusual in *Campylobacter*, which more frequently utilizes various inactivating enzymes (Fabre et al., 2018). The involvement of these *16S rRDNA* mutations in gentamicin resistance remains to be demonstrated experimentally. The emergence of resistance to ertapenem also reflects the high susceptibility to mutation of the strains found in the two patients in our study. Roa-Bautista et al. also described carbapenem resistance but not the associated mechanism (Roa-Bautista et al., 2023). We identified one ertapenem- and meropenem-resistant *C. coli* isolate in patient #2 (2C04). Resistance is probably due to a motif duplication in the major porin PorA sequence, although its actual involvement in carbapenem resistance has yet to be demonstrated experimentally. Like other surface proteins, PorA may be subject to strong selection pressure both from the host response and from antibiotics, perhaps particularly after prolonged repetitive treatment with carbapenems. No significant PorA SNPs were identified in the other ertapenem-resistant only *C. coli* isolates from patient #1 (1C04, 1C05, 1C06, 1C09), with ertapenem MICs ranging from 1.5 to 2 mg/L. The reasons for this remain unclear. The emergence of carbapenem resistance in the context of XLA infection in *C. jejuni* has been described previously (Nunes et al., 2023; Zhuo et al., 2024), with the appearance of mutations or insertions within the PorA sequence. The emergence of post-treatment resistance to carbapenems, resulting from a deletion in PorA at position A71, has also been observed in a *C. coli* bacteremia isolate from a patient with God’s syndrome (Maurille et al., 2024). Fecal transplantation may be used to treat patients with CVID or XLA. Although this proved unsuccessful for patient #2, it may benefit patients with debilitating digestive symptoms associated with bacteremia.

A major limitation of this study was that we did not have access to the strains present in the digestive reservoir of the two patients concerned. The bacteremia episodes studied were generally not associated with intestinal disorders, except for patient #1 who had diarrheal episodes in 2008 and 2019 (the *C. jejuni* strains were not conserved). We cannot determine the presence or absence of strains with distinct resistance profiles from previous episodes. In such patients, it would be interesting to combine PCR and culture of *Campylobacter* in the intestine with simultaneous hemoculture. This could be carried out in the absence of digestive symptoms to assess the importance of chronic digestive carriage associated with occult bacteremia, or during febrile epidemics to confirm digestive translocation. Without digestive isolates, the hypothesis of microevolution versus reinfection remains therefore partly speculative. We present observational associations of in-vivo selection, but we cannot exclude co-existing resistant populations in the gut, undetected colonization events, or mixed strain reservoirs. Finally, analyzing recombination events could be another way to compare the isolates described in the present study since they may be a major driver of genetic diversity in *Campylobacter* spp. (Wilson et al., 2009).

## Conclusion

Our study illustrates the difficulties involved in treating *Campylobacter* infections in patients with CVID for whom repetitive treatments targeting the antibiotic sensitivity profile appear ineffective in eliminating the digestive reservoir. We show that these strains may be highly subject to mutations, allowing them to counteract the antibiotics used and evade the host response against a background of recurrent bacteremia. This study emphasizes the need for integrated clinical and genomic surveillance.

## Data Availability

Genome data are available from the European Nucleotide Archive (ENA) BioProject PRJEB87827. ENA accessions for each clinical isolate are shown in Supplementary Table 1.

## Supplementary material

Supplementary Table 1 Complete metadata for isolates of *C*. *coli* and *C*. *jejuni* from both patients. Additional data for the clinical isolates that are not shown in Table 1: reads accession numbers (ENA), sequencing information (sequencer used, genome size, contig number, number of coding sequences, and GC%), cgMLST result using Oxford scheme v1, and full carbapenem susceptibility data for ertapenem, meropenem, and imipenem.

Supplementary Figure 1 Protein structure modeling of PorA for isolate 2C03 (susceptible to carbapenems) and 2C04 (resistant to ertapenem and meropenem). The AlphaFold protein structure prediction tool was used to display the PorA three-dimensional structure for isolates 2C03 and 2C04. The carbapenem-resistant isolate 2C04 exhibited a DNAIDGL motif duplication in the protein sequence at position 139; this may obstruct the PorA ion channel.
