# Supplementary figures and images for: Antimicrobial use on Campylobacter revealed by next-generation sequencing in patients with common variable immunodeficiency
Source: Front Microbiol. 2026 Apr 1;17:1750824. doi: 10.3389/fmicb.2026.1750824 (PMC13079058; doi:10.3389/fmicb.2026.1750824)

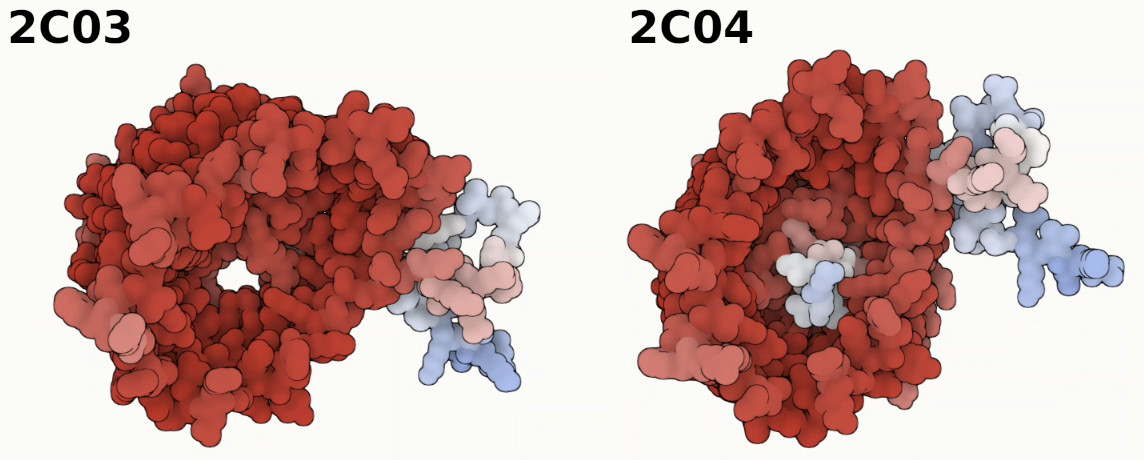

Supplement: Supplementary Figure 1 — Protein structure modeling of PorA for isolate 2C03 (susceptible to carbapenems) and 2C04 (resistant to ertapenem and meropenem). The AlphaFold protein structure prediction tool was used to display the PorA three-dimensional structure for isolates 2C03 and 2C04. The carbapenem-resistant isolate 2C04 exhibited a DNAIDGL motif duplication in the protein sequence at position 139; this may obstruct the PorA ion channel. [file Image_1.JPEG]
